# Design and effectiveness evaluation of mirror myoelectric interfaces: a novel method to restore movement in hemiplegic patients

**DOI:** 10.1038/s41598-018-34785-x

**Published:** 2018-11-12

**Authors:** Andrea Sarasola-Sanz, Nerea Irastorza-Landa, Eduardo López-Larraz, Farid Shiman, Martin Spüler, Niels Birbaumer, Ander Ramos-Murguialday

**Affiliations:** 1 Institute of Medical Psychology and Behavioral Neurobiology, University of Tübingen, Tübingen, Germany; 2 International Max Planck Research School for Cognitive and Systems Neuroscience, Tübingen, Germany; 3 Tecnalia, San Sebastián, Spain; 4 IKERBASQUE, Basque Foundation for Science, Bilbao, Spain; 5 Department of Neurology, Campus Mitte, Charité – Universitätsmedizin Berlin, Berlin, Germany; 6 Department of Computer Engineering, Wilhelm-Schickard-Institute, University of Tübingen, Tübingen, Germany; 7 Wyss Center, Geneve, Switzerland

## Abstract

The motor impairment occurring after a stroke is characterized by pathological muscle activation patterns or synergies. However, while robot-aided myoelectric interfaces have been proposed for stroke rehabilitation, they do not address this issue, which might result in inefficient interventions. Here, we present a novel paradigm that relies on the correction of the pathological muscle activity as a way to elicit rehabilitation, even in patients with complete paralysis. Previous studies demonstrated that there are no substantial inter-limb differences in the muscle synergy organization of healthy individuals. We propose building a subject-specific model of muscle activity from the healthy limb and mirroring it to use it as a learning tool for the patient to reproduce the same healthy myoelectric patterns on the paretic limb during functional task training. Here, we aim at understanding how this myoelectric model, which translates muscle activity into continuous movements of a 7-degree of freedom upper limb exoskeleton, could transfer between sessions, arms and tasks. The experiments with 8 healthy individuals and 2 chronic stroke patients proved the feasibility and effectiveness of such myoelectric interface. We anticipate the proposed method to become an efficient strategy for the correction of maladaptive muscle activity and the rehabilitation of stroke patients.

## Introduction

There is extensive evidence that the motor system coordinates muscle activations through a superposition of activations of different groups of muscles as single units (i.e. superposition of muscle synergies) that are specified at the spinal or brainstem level^1–4^. Further investigation into the nature and characteristics of the electromyographic (EMG) activity of stroke patients has led to the discovery of abnormal patterns of muscle activations or synergies that may result from maladaptive compensatory strategies^5–7^. After a stroke, cortical and/or subcortical damage interferes with the flow of descending signals to the spinal cord, which yields a disrupted recruitment of muscle synergies and so, a pathological muscle coordination^8,9^. Furthermore, it has been found that the preservation of muscle synergies has a positive correlation with hand functionality in severely paralyzed patients with intact sensorimotor cortex^7^. It follows that this abnormal movement coordination might constitute the primary source of movement dysfunction (spasticity and muscle weakness being secondary^10^) and that the recovery of healthy synergies may be to some extent, linked to the improvement of the upper limb motor function.

Physical therapy is the traditionally accepted rehabilitation method for stroke patients. However, in recent years, robot-aided training has become one of the most widely explored rehabilitation strategies for this type of patients. It allows repetitive, functional, meaningful, intensive and challenging training, which has been proven to promote neuroplasticity and motor learning^11–13^. Various control signals and strategies have been used for robot-aided rehabilitation therapies. Some devices adapt the provided assistance level based on participant’s interaction forces^14–16^. Others allow free movements for a fixed time and then move the hand if the participant is not able to complete the task^17–19^. Myoelectric interfaces are often proposed in rehabilitation therapies for the control of body actuators such as wearable robots or prosthesis^20,21^.

A myoelectric interface is a system that decodes the intention of the patient from the electromyographic (EMG) activity of the paretic limb and sends the corresponding commands to the body actuator. This allows patients to generate volitional movement through their normal cortico-spinal pathways and provides them with feedback (e.g. proprioceptive and visual feedback), establishing a closed-loop system that promotes learning. These systems also encourage the active participation of the patient and they can improve muscle coordination and strength and reduce spasticity after training^22^. However, this raises the question of whether it is possible to find decodable EMG activity in hemiplegic patients of any impairment level. Some studies reported a decoding accuracy between 36.7% and 96.1% with 4–20 movement classes using the EMG of the paretic upper limb in mild to severely impaired stroke patients^23–25^. Recently, 46% of the 41 severe chronic stroke patients enrolled in a 1-month brain-machine interface (BMI) training study regained decodable EMG activity (accuracy > 65%)^26^. Interestingly, in some cases, decodable EMG was found even in the absence of movement of the paretic arm. These results opened up the door of EMG-based rehabilitation therapies to severely affected stroke patients, who cannot benefit from many other rehabilitation techniques^27^, in which residual movement of the paretic limb is necessary.

Among the existing EMG-based control strategies, a simple approach is to trigger a pre-programmed assistive movement when the EMG amplitude goes over a threshold^28^. Other controllers provide assistive forces proportional to the EMG amplitude of the impaired limb^22,29,30^. More complex myoelectric control interfaces are based on classification techniques (i.e. mapping the EMG into predefined discrete movements) or on continuous trajectory-decoding strategies (i.e. mapping the EMG into velocity of the movement). Classification techniques have been investigated for the post-stroke rehabilitation showing encouraging but still limited results^23–25,31^. On the other hand, continuous decoding strategies offer a more intuitive and natural myoelectric control, which allows for a richer therapy with a wider range of trained and untrained movements, and facilitates a less effortful and fine control, thus leading to a better training and probably by extension, to motor recovery too^32–41^. However, up to now very few studies used such methods to this end^38–40^ and they are limited to neurologically intact subjects or allow the simultaneous control of up to 3 degrees of freedom (DoFs) of the upper limb through a virtual reality interface^38^ or a cursor on a screen^39^. Liu *et al*.^40^, also used an upper-limb exoskeleton to record EMG activity and kinematics and compute the offline decoding performance. However, their system allows the simultaneous control of 3 DoFs only (angle of the shoulder, angle of the elbow and wrist), which limits the possibility of performing a functional training including the hand joints. Therefore, the development of a rehabilitation system that allows a continuous (i.e. trajectory) and reliable myoelectric control of several proximal and distal DoFs of the upper limb simultaneously still remains a challenge.

Myoelectric interfaces for rehabilitation aim at activating neuroplastic mechanisms that reshape muscle activity and lead to motor learning, and eventually to motor function restoration. However, it could be argued that using a myoelectric decoder calibrated with paretic EMG data (i.e. an ipsilateral myoelectric decoder), could indeed reinforce the existence and maintenance of pathological synergies (i.e. promote “bad” neuroplasticity). Cesqui *et al*.^25^ tackled this problem by building a model of healthy muscle patterns from data collected on 9 healthy participants and using it to classify the paretic EMG of stroke patients into reaching movements towards 4 different positions. Although this is the only approach designed to enhance the recovery of healthy activations, it is limited by: (i) the necessity of forming a large database of EMG activity from healthy subjects to generalize to the specific anatomical and neurophysiological characteristics of each patient and (ii) the fact that the decoder was confined to the classification of the EMG activity into four discrete movements, involving only proximal joints.

Here, we propose a new upper limb rehabilitation paradigm for stroke patients that overcomes the aforementioned limitations and puts special emphasis on the recovery of healthy and natural muscle activation patterns or synergies. We present a novel myoelectric interface that decodes the patient’s EMG into the direction and speed of a 7-DoF upper limb exoskeleton during functional tasks. Previous evidence^8,9^ indicates that there are no substantial inter-limb differences in the synergy structure of healthy individuals. Hence, our myoelectric decoder, which we will refer to as the mirror myoelectric decoder, is calibrated with data mirrored from the healthy upper limb of the patient and serves as a reference model and a learning tool for him/her to reshape his/her muscle activation patterns. We present and evaluate the effectiveness of the proposed rehabilitation system in 8 healthy individuals and 2 chronic stroke patients.

## Methods

### Novel rehabilitation paradigm

Our novel motor rehabilitation paradigm is designed to reshape the muscle activation patterns of chronic stroke patients. It is intended to be used as part of a closed-loop rehabilitation system. In our setup, the IS-MORE 7-DoF robotic exoskeleton (Tecnalia, San Sebastian, Spain) acts as the body actuator that provides the patient with proprioceptive and visual feedback during functional task training.

**Figure 1:**
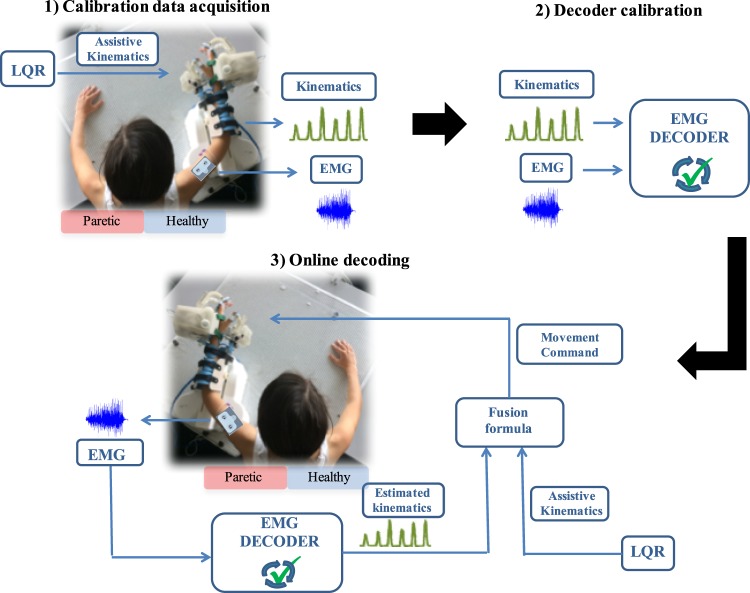
Steps to follow to build and use a mirror myoelectric decoder: (1) Calibration session: EMG and kinematic data are recorded from the healthy upper limb during different functional movements with the exoskeleton. (2) The recorded data is used to calibrate a mirror EMG decoder (i.e. build a healthy EMG-kinematics mirror model). (3) The EMG activity recorded from the paretic limb during the real-time phase is fed to the mirror decoder. The latter is able to map the input EMG signal into the corresponding kinematics based on the mirror myoelectric model of healthy activity. The estimated kinematics are sent as control commands to the exoskeleton, which drives the movement of the paretic upper limb of the patient, providing him/her with visual and proprioceptive feedback.

The steps to build and utilize the rehabilitation system are summarized in Fig. 1. First, EMG and kinematic data is acquired from the healthy upper limb of the hemiplegic patients while they perform a series of functional tasks with an exoskeleton. This data is utilized to build a subject-specific model of healthy EMG-to-kinematics mirrored to be used by the paretic arm (i.e. the DoFs that have opposite sign for left and right arms are flipped before building the model). Then, this model would be used to provide feedback about the EMG of the paretic upper limb. Thus, it would serve as a learning tool for the patient to reproduce the same correct healthy muscle activation patterns that have been mirrored to the paretic side. During the real-time operation, patients would try to perform similar functional tasks while wearing the exoskeleton and having EMG electrodes placed over the equivalent muscles on the impaired limb. In this phase, the myoelectric interface would receive the paretic EMG signals as input and would predict the corresponding kinematics based on the mirror model. The predicted kinematics would determine the movement of the exoskeleton. In this way, this closed-loop system would provide the patient with visual, haptic and proprioceptive feedback about his/her muscle activations. For example, if the patient produces uncoordinated activation patterns the exoskeleton would deviate from the intended trajectory or move at a lower speed (depending on the control strategy). Hence, patients would have to modulate their EMG activity to produce the correct patterns that would bring the exoskeleton towards the target position. Thus, the purpose of this novel rehabilitation paradigm is to provide patients with feedback about the appropriate recruitment of their muscles, rather than only interpreting their motion intention independently of whether the EMG shows correct activation patterns for the intended movement or not.

### Study design

One experiment in 8 able-bodied individuals and one proof of concept in 2 chronic stroke patients were performed to investigate the optimal parameters and validate the effectiveness of the system. Each of them was performed under slightly different conditions (see Table 1) and served to analyze various features of the system:

**Table 1:** Design of the two experiments. Differences and similarities between them.

Sessions	Experiment Healthy Participants			Experiment Healthy Participants							Experiment Stroke Patients	
	S1	S2	S3	S4		S5		S6		S7	S1	S2
Purpose	1. Influence of the session-to-session and arm-to-arm transfers on the decoding performance 2. Generalization ability of mirror decoders			Compliance conditions for the calibration session							Preliminary tests of the effectiveness of the system in stroke patients	
Participants	8 healthy individuals			5 healthy individuals							2 chronic stroke patients	
Arm side	Right	Right	Left	Right		Left		Left		Right	Right (healthy)	Left (paretic)
Tasks	T1, T2, T3, T4 + Free movements			T1, T2, T3							T1, T2 (Patient 1); T1, T2, T3, T4 (Patient 2)	
Compliance	Active			Active		Active		Compliant		Compliant	Compliant	

#### Studying the influence of the session-to-session and arm-to-arm transfers on the decoding performance

Factors such as varying upper limb positions, impedance changes, electrode shifts across days, and the EMG activity pattern disparity across arms might dramatically alter the decoding accuracy. We evaluated the influence of these factors on the performance of within- and across-sessions and -arms decoders.

#### Generalization ability of the mirror decoder

As new tasks or movement with bigger range of motion could be included during the therapy, we assessed the decoder’s ability to decode untrained movements, which were not present in the calibration data. We compared several task-specific decoders with a single general decoder, considering properties such as practicality and accuracy.

#### Optimal conditions for calibration data recording

Two conditions were considered to record the calibration data: (i) *Active*: the motors of the exoskeleton were off and users had to overcome the friction and weight of the robot (i.e., the robot was used as a kinematic sensing device only), (ii) *Compliant:* the motors of the exoskeleton on, being the exoskeleton the one driving the fully-assistive movement. The users were explicitly asked to follow it trying to naturally activate their muscles and to avoid counteracting or forcing the movement of the exoskeleton.

#### Proof of concept with chronic stroke patients

This experiment was designed to test the offline performance of the general mirror decoder in chronic stroke patients.

### Experimental Protocol

**Figure 2:**
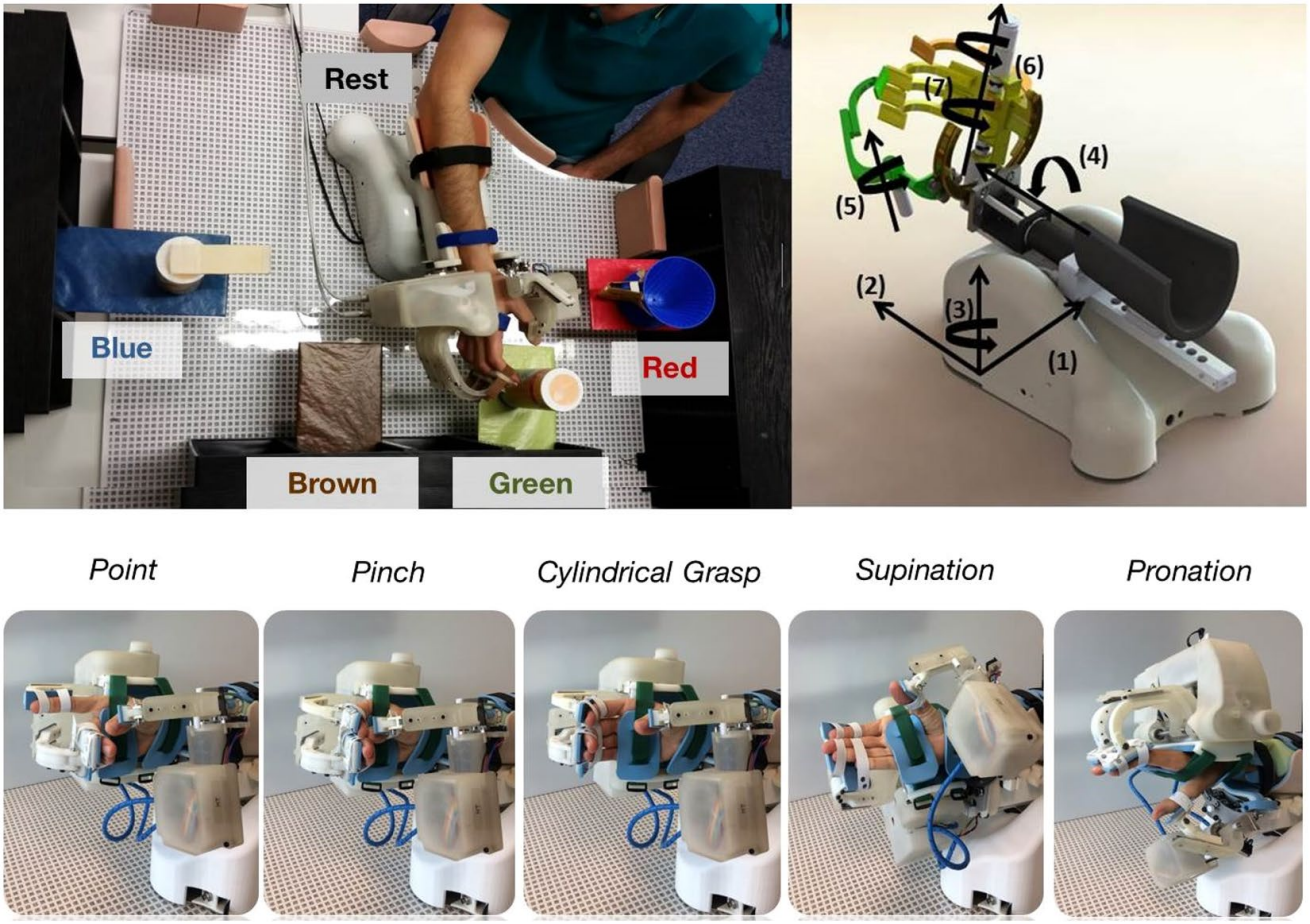
Experimental setup and tasks performed: **(a)** Workspace where the tasks were performed. “Pos 1–4” indicate the four colored targets and “Rest” the predefined rest position in which the trials started and ended. The three objects the participants had to interact with are also shown on top of the target shelves. **(b)** A model of the IS-MORE 7-DoF exoskeleton and the degrees of freedom on which it allows the movement: (1) and (2): translation of the forearm (2 DoFs); (3): rotation of the forearm (1 DoF); (4): wrist pronation-supination (1 DoF); (5): extension-flexion of the thumb (1 DoF), (6): the index (1 DoF) and (7): the group of middle-ring-pinky fingers (1 DoF). **(c)** Wrist and hand movements performed during the experiments.

None of the healthy participants presented any neuromuscular disorder. All healthy participants and patients gave written informed consent to the procedures as approved by the ethics committee of the Faculty of Medicine of the University of Tübingen, Germany. All the experiments were performed according to the guidelines of the University of Tübingen and constitute a proof of concept for the clinical trial registered on the 5^th^ of February of 2018 with the registration ID: DRKS00013926. Healthy participants and patients were asked to sit and perform a series of functional tasks while wearing an upper limb exoskeleton either on their right or left arm (see Table 1). The IS-MORE exoskeleton allowed movements in 7 DoFs, including proximal (upper- and forearm) and distal (fingers and wrist) segments of the arm. The setup consisted of four coloured targets located on both sides and in front of the participant, and a 70 × 50 cm mat on top of which the exoskeleton was placed and the movements were executed (see Fig. 2). Healthy participants and patients were instructed by means of auditory cues to perform up to four different functional tasks, which always started and ended at a predefined rest position that kept the arm and the hand in a relaxed configuration:

- T1) Reaching task. This task 1 consisted of reaching movements from the rest position towards each of the four different targets and then back to the initial rest position.
- T2) Static grasping task. Participants were asked to keep their arm still at the rest position while performing five different movements that involved finger and wrist joints: pinch grip, cylindrical grasp, pointing, supination and pronation of the wrist (see Fig. 2).
- T3) Double reaching + pointing task. First, participants had to reach one target while pointing at it, then move to another target while keeping the pointing gesture and finally come back to the initial rest position.
- T4) Double reaching + object grasping task. Participants were instructed to reach a target, grab the object placed at that position, bring it to a different target position in which they had to deposit it and then come back to the initial rest position. Three objects of different sizes and shapes, which required performing a pinch grip, a key grip or a cylindrical grasp to take them, were presented to the participants.

Each of the four tasks was divided in 5 blocks of 40, 42, 10 and 22 trials, respectively. For the experiment with stroke patients, sessions were notably shorter ranging from 2–4 blocks depending on the self-reported fatigue and containing 8, 10, 6 and 6 trials each of the four tasks respectively. All participants could rest for a few minutes between blocks and inter-trial intervals of 2–3 secs were included to avoid fatigue. Tasks T1-T4 were always performed in this order, completing all the blocks of each task before moving onto the blocks of the next task type.

#### Healthy participants

Eight healthy individuals (3 females, 5 males, age: 20–28, all right-handed) underwent three sessions (S1: right upper limb; S2: right upper limb; S3: left upper limb) on distinct days.

The four functional tasks explained above were included in this study. Additionally, at the end of each session subjects were asked to move their upper limb freely around the whole workspace (Free-Movement Task) during 3 minutes, without any time constraint, predefined task or trial structure. These three sessions S1-S3 were used to analyze the session-to-session and arm-to-arm influence on the decoding performance as well as the generalization ability of task-specific and general decoders (analyses 1 and 2).

It is important to emphasize that in these three sessions the exoskeleton was passive (i.e. *Active* condition from the user perspective, exoskeleton motors were off) and hence, subjects had to exert sufficient force to move the exoskeleton towards the required target position without any type of assistance.

In order to investigate the optimal way of recording the calibration data (analysis 3), five of the eight healthy participants (3 females, 2 males, age: 21–28, all right-handed) underwent four more sessions (S4: right upper limb; S5: left upper limb; S6: left upper limb; S7: right upper limb) on separate days.

In these extra sessions S4-S7, the first three tasks of the set of functional tasks described above (T1, T2 and T3) were included. In sessions S4 and S5, just as in the previous S1-S3, no assistance was provided for the movement of the exoskeleton (i.e. *Active* condition). In sessions S6 and S7 participants performed the tasks with the exoskeleton actively moving their arm and participants following that exact movement (i.e. *Compliant* condition).

During the active condition sessions, participants could execute the movements at their own pace but within a given time interval (5 secs for task 1 trials, 4 secs for task 2 trials, 8 secs for task 3 trials and 8 secs for task 4 trials). During the compliant sessions, instead, the direction and speed of the movement was predefined and customized for each participant according to their range of motion and kept constant among the various trials of each task.

#### Stroke patients

Two chronic stroke patients (2 males, Patient 1 (P1): age = 47 years, time since stroke = 5 years, moderate left hemiparesis according to^42^, modified upper limb FMA = 87/114 and combined hand and arm FMA = 29/54; Patient 2 (P2): age = 62 years, time since stroke = 2 years, severe left hemiparesis, modified upper limb FMA = 59/114 and combined hand and arm FMA = 7/54) participated in this experiment consisting of two sessions (S1: healthy upper limb; S2: paretic upper limb) on different days. In both sessions, movements were executed under *Compliant* conditions. Regarding the tasks, P1 performed 4 blocks (healthy arm) and 1 block (paretic arm) of the first two functional tasks described above (T1-T2) while P2 carried out a training including 2 blocks (healthy and paretic arms) of the four tasks (T1-T4).

### Data collection and processing

In healthy participants, EMG activity was recorded at 2500 Hz (Brain Products GmbH, Germany) from 10 standard bipolar electrodes with an inter-electrode distance of 2.2 cm (Myotronics-Noromed, USA) over: (1) the abductor pollicis longus, (2) the extensor carpi ulnaris, (3) the extensor digitorium, (4) the flexor carpi radialis, palmaris longus and flexor carpi ulnaris, (5) the pronator teres, (6) the long head of the biceps, (7) the external head of the triceps, (8) the anterior portion of the deltoid, (9) the lateral portion of the deltoid and (10) the posterior portion of the deltoid over the teres minor and infraespinatus muscles. Kinematics were recorded at 18 Hz (details in^43,44^).

EMG data was band-pass filtered at 10–500 Hz and power-line notch filtered at 50 Hz. Seven time-domain features (Mean of absolute values, Variance, Waveform Length, Root-mean-square value, Willison Amplitude (WAMP), Zero crossing (ZC) and Slope sign changes (SSC))^41,45^ were extracted from each EMG channel in windows of 200 ms producing a set of 70 EMG features. This set of EMG features was down-sampled to 18 Hz and synchronized with the kinematic data, which was low-pass filtered at 1.5 Hz.

The EMG signal was normalized as in an online setup, using the mean and standard deviation computed in a 60sec-window of past samples. The estimated kinematics were smoothed with a weighted moving average filter (backwards window of 550 ms and linearly decreasing weights) to avoid a jerky and unstable control of the exoskeleton.

All the EMG channels were included in the control of each DoF, independently of them being directly, indirectly or not related at all to the movement of that specific DoF. Since this rehabilitation approach relies on the feedback given to the patients as a way of teaching them to recover healthy muscle activation patterns, including all the muscles in the control of each DoF could help them to avoid compensatory activations of non-related muscles.

For patients, EMG data was recorded at 1000 Hz. P2 used the same 10 bipolar electrodes as healthy participants. However, for the patient P1 the 5 bipolar electrodes over the extensor and flexor muscles of the forearm were substituted by two high-density arrays (Tecnalia, Spain) including 24 (6 × 4) monopolar electrodes each with an inter-electrode distance of 1.35 cm (horizontal) and 2 cm (vertical). These monopolar channels were bipolarized summing up to 100 channels and no dimensionality reduction was applied.

The three time-domain threshold-dependent features (i.e. WAMP, ZC and SSC) were removed from the processing to avoid any inaccuracies arising from the different EMG amplitude across arms. Instead, the logarithm of the variance was included as the fifth component of the feature set.

### Decoding schemes and algorithm

We compare ipsilateral (within arm) and mirror (across arms) decoding schemes:

#### Ipsilateral decoding schemes

- *Within-session decoder (WS):* This decoder was calibrated and tested with data from the same session and arm, following a 5-fold cross-validation^43^.
- *Session-to-session decoder (SS):* This decoder was calibrated with data from one session and tested with data from a different session in which the movements were performed with the same arm.
- *Re-calibrated session-to-session decoder (RSS)*: About 10 minutes of data collected at the beginning of the new session together with the data from the previous session were used to calibrate a new (“re-calibrated”) session-to-session (SS) decoder^43^. The testing was performed on the remaining data of the new session.

#### Mirror decoding schemes

- *Task-specific arm-to-arm decoder (TSAA):* This decoder was task specific and was calibrated with data from a specific task during a session(s) with one of the arms and tested during that same task from a different session using the other arm.
- *General arm-to-arm decoder (GAA):* This decoder was also calibrated and tested with data from different arms. However, this was the only decoder that gathered all the performed tasks in one decoder and thus, was not task-specific.

**Figure 3:**
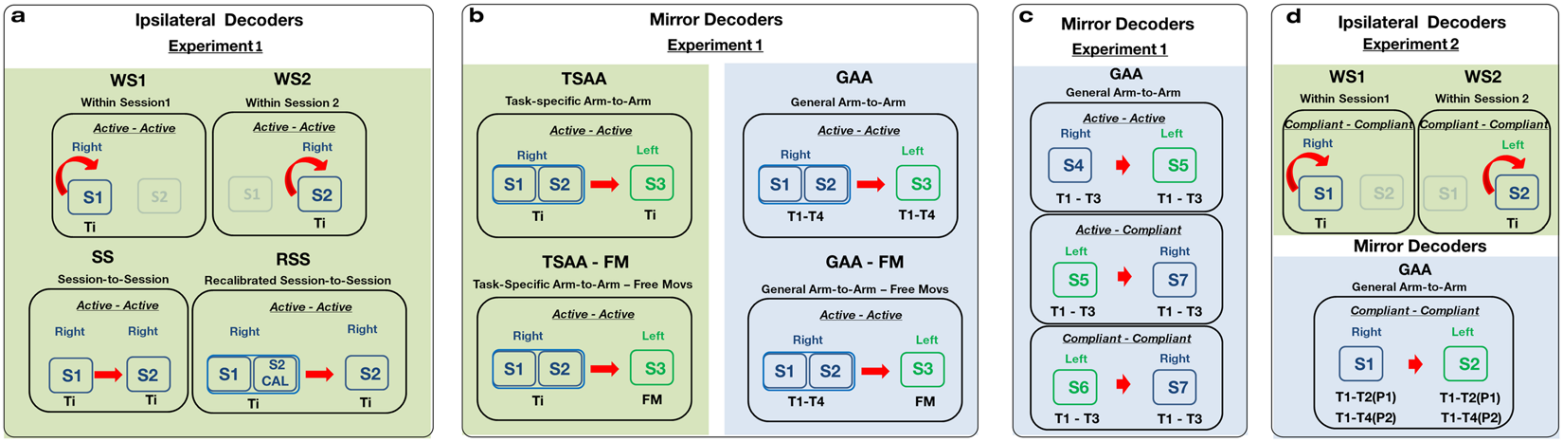
Ipsilateral and mirror decoders built with data from the two experiments: **(a)** Ipsilateral and **(b)** mirror decoders from sessions S1-S3 of the experiment with healthy participants. **(c)** Mirror decoders from sessions S4-S7 of the experiment with healthy participants. **(d)** Ipsilateral and mirror decoders from the experiment with stroke patients. For the four diagrams, the information to the left of the red arrow refers to the calibration data and the one the arrow is pointing to, to the testing data. The green background color indicates that the decoders are task-specific, whereas the blue background color signifies that the decoders where general. The tasks that were used for calibrating and testing are displayed at the bottom left and right of the squared box of each decoder respectively. For the task-specific decoders, Ti indicates that a separate decoder was built for each of the four tasks Ti, i = 1–4. For the general decoders, Tk-Tj means that all the tasks from k to j were used for calibrating and/or testing a decoder. FM stands for the free movement task. The mirror decoder of the experiment with stroke patients specifies the tasks employed for each of the patients P1 and P2. The “Left” and “Right” labels specify which limb the participants wore the exoskeleton on during that session. Finally, the “*Active-Active*”, “*Active-Compliant*” and “*Compliant-Compliant*” labels inform about the compliance conditions under which the “calibration- testing” data was recorded.

All the aforementioned ipsilateral and mirror decoders were subject-and DoF-specific. Figure 3 illustrates all the variations of the decoding schemes that were analyzed in this study.

The ridge regression algorithm was selected to predict the output kinematics of each DoF from the input EMG features. Although this linear algorithm has several limitations (e.g. various activation patterns could lead to the same kinematics), it allows the simultaneous and proportional control over multiple DoFs, it was also proven to outperform other methods such as the regular Kalman filter^43^ and to perform similar to non-linear regression methods online^46^, and it penalizes the co-activation of agonist-antagonist pairs, which is an important aspect for stroke rehabilitation. The optimum value of the regularization parameter λ for the WS schemes was found in a nested cross-validation loop using a grid search of values in the range [10^−7^, 10^7^]. However, for the rest of the decoding schemes λ was fixed at 10^4^, chosen experimentally^47^.

### Statistics

The EMG-decoding was computed offline in a pseudo-online manner (i.e. streaming the data into the decoder as in a real-time scenario). The decoding performance was measured by comparing the smoothed kinematics predicted from the EMG activity and the smoothed kinematics recorded with the exoskeleton. The Pearson correlation coefficient (CC) and the normalized root-mean-square error (NRMSE) were used as performance metrics. The overall performance of each decoder was computed as the average over the 7-DoFs, all the tasks and all the participants of each experiment. The α-level for all the statistical tests was set to 0.05.

The statistical tests applied to the four analyses described above are:

1. After checking for the normality of the data distribution, all the ipsilateral and mirror decoders utilized to study the session and arm transfer influence (Fig. 3a,b) were compared with a one-factor repeated measures analysis of variance (ANOVA), being the factor the decoding scheme. Significant results were followed by post-hoc pairwise comparisons using paired t-tests with Bonferroni correction.
2. A paired t-test was computed to compare the generalization ability of the mirror decoders tested only in the unrestricted free movements (FM) (i.e. TSAA-FM vs. GAA-FM of Fig. 3b).
3. The performance of the general decoders calibrated and tested under Active or Compliant conditions (Fig. 3c), were compared with a 1-factor (compliance condition) ANOVA test, followed by Bonferroni-corrected post-hoc comparisons.
4. Finally, the ipsilateral decoder of the healthy arm, and the ipsilateral and general mirror decoders of the paretic arm were tested in stroke patients (Fig. 3d) and their performance was reported for each patient separately.

## Results

### Session-to-session and arm-to-arm transfers’ influence

**Figure 4:**
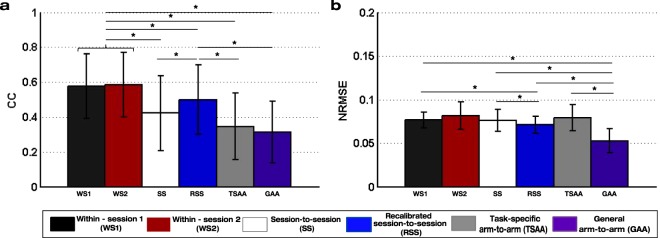
Performance values of the ipsilateral and mirror decoders from sessions S1-S3 of the experiment with healthy participants: (**a**) Correlation coefficient (CC) and (**b**) normalized root-mean-square error (NRMSE) mean and standard deviation values of the ipsilateral decoders: within-session (WS1 and WS2), session-to-session (SS) and recalibrated session-to-session (RSS); and the mirror decoders: task-specific arm-to-arm (TSAA) and general arm-to-arm (GAA) decoders of the experiment with healthy participants. The asterisks show significant (p < 0.05) differences between decoders.

The session-to-session and arm-to-arm transfer influence due to factors such as electrode shift and inter-limb variability was assessed by comparing the performance of ipsilateral and mirror decoders (Fig. 3a,b). The performance for each of them is presented in Fig. 4. The ANOVA showed significant differences between these decoders for both the CC (p < 10^−6^) and NRMSE (p = 4.0·10^−6^). Subsequent Bonferroni-corrected pairwise comparisons (see Table 2) showed that the within-session and recalibrated decoders (WS1, WS2 and RSS) outperformed the other three (SS, TSAA and GAA) in terms of CC. However, no significant difference was found between the CC values of the session-to-session (SS) and the two mirror (TSAA and GAA) decoders. The error of the general arm-to-arm decoder (GAA) was significantly lower than all the others except the within-session decoder of session S2 (WS2).

**Table 2:** Bonferroni corrected p- values of the ANOVA test comparing the correlation coefficient (CC) and normalized root-mean-square error (NRMSE) of the decoders from sessions S1-S3 of the experiment with healthy participants.

	Within-session 2 (WS2)	Session-to-session (SS)	Recalibrated session-to-session (RSS)	Task-specific arm-to-arm (TSAA)	General arm-to-arm (GAA)
WS1	*p*_*CC*_ = 1.000 *p*_*NRMSE*_ = 1.000	***p***_***CC***_ ** = 3.2·10** ^**−4**^ ***(CC***_***WS*1**_ ** > ** ***CC***_***SS***_ **)** *p*_*NRMSE*_ = 1.000	***p***_***CC***_ ** = 0.005** **(** ***CC***_***WS*1**_ ** > ** ***CC***_***RSS***_ **)** ***p***_***NRMSE***_ ** = 0.032** ***(NRMSE***_***WS*1**_ ** > ** ***NRMSE***_***RSS***_ **)**	***p***_***CC***_ ** = 1.0 ·10** ^**−5**^ ***(CC***_***WS*1**_** > *CC***_*TSAA*_) *p*_*NRMSE*_ = 1.000	***p***_***CC***_ ** = 1.7 ·10** ^**−5**^ ***(CC***_***WS*1**_ ** > ** ***CC***_***GAA***_ **)** ***p***_***NRMSE***_ ** = 2.1 ·10** ^**−5**^ ***(NRMSE***_***WS*1**_ > ***NRMSE***_***GAA***_**)**
WS2		***p***_***CC***_ ** = 1.5 ·10** ^**−5**^ ***(CC***_***WS*2**_ ** > ** ***CC***_***SS***_ **)** *p*_*NRMSE*_ = 1.000	***p***_***CC***_ ** = 4.7·10** ^**−4**^ ***(CC***_***WS*2**_ ** > ** ***CC***_***RSS***_ **)** *p*_*NRMSE*_ = 1.000	***p***_***CC***_ ** = 2.5 ·10** ^**−5**^ ***(CC***_***WS*2**_ ** > ** ***CC***_***TSAA***_ **)** *p*_*NRMSE*_ = 1.000	***p***_***CC***_ ** = 9.2 ·10** ^**−5**^ ***(CC***_***WS*2**_ ** > ** ***CC***_***GAA***_ **)** *p*_*NRMSE*_ = 0.157
SS			***p***_***CC***_ ** = 0.003** ***(CC***_***SS***_ ***<CC***_***RSS***_ **)** ***p***_***NRMSE***_ ** = 0.023** ***(NRMSE***_***SS***_ ** > ** ***NRMSE***_***RSS***_ **)**	*p*_*CC*_ = 0.057 *p*_*NRMSE*_ = 1.000	*p*_*CC*_ = 0.05 ***p***_***NRMSE***_ ** = 7.2 ·10** ^**−4**^ ***(NRMSE***_***SS***_ > ***NRMSE***_***GAA***_**)**
RSS				***p***_***CC***_ ** = 1.1 ·10** ^**−4**^ ***(CC***_***RSS***_ ** > ** ***CC***_***TSAA***_ **)** ***p***_***NRMSE***_ ** = 0.207**	***p***_***CC***_ ** = 4.2 ·10** ^**−4**^ **(** ***CC***_***RSS***_ ** > ** ***CC***_***GAA***_ **)** ***p***_***NRMSE***_ ** = 0.002** ***(NRMSE***_***RSS***_ ** > ** ***NRMSE***_***GAA***_ ***)***
TSAA					*p*_*CC*_ = 0.139 ***p***_***NRMSE***_ ** = 1.7 ·10** ^**−5**^ ***(NRMSE***_***TSAA***_ ** > ** ***NRMSE***_***GAA***_ ***)***

In bold all p-values < 0.05 (significance level).

### Generalization ability of the mirror decoding schemes

**Figure 5:**
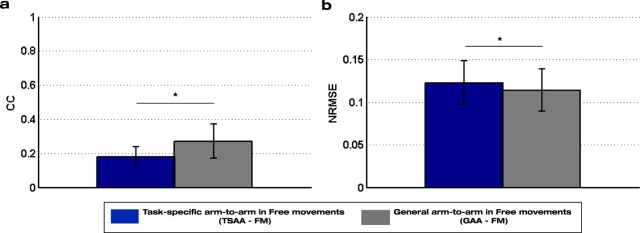
Performance values of the mirror decoders from sessions S1-S3 of the experiment with healthy participants tested on free movements: (**a**) Correlation coefficient (CC) and (**b**) normalized root-mean-square error (NRMSE) mean and standard deviation values of the task-specific arm-to-arm (TSAA-FM) and general arm-to-arm (GAA-FM) decoders tested on the three minutes of free movements of the experiment with healthy participants. The asterisks show significant (p < 0.05) differences between decoders.

Table 2 shows the lower error (p = 1.7·10^−5^) of the general mirror decoder GAA compared to the equivalent task-specific one TSAA, when tested in tasks T1-T4 that were included in the calibration set. Additionally, these two mirror decoders were tested during free movements (TSAA-FM and GAA-FM) and their comparison confirms the significantly better generalization ability of the general decoder compared to the task-specific one (CC: p = 0.012; NRMSE: p = 0.017) (see Fig. 5).

### Optimal conditions for calibration data recording

**Figure 6:**
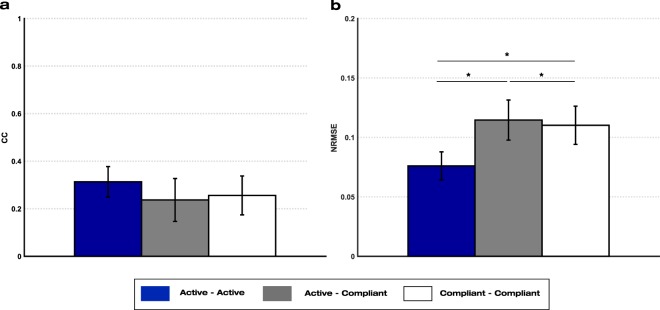
Performance values of the mirror decoders from sessions S4-S7 of the experiment with healthy participants: (**a**) Correlation coefficient (CC) and **(b)** normalized root-mean-square error (NRMSE) mean and standard deviation values of the general arm-to-arm (GAA) decoder with different compliance conditions for the calibration and testing data. The asterisks show significant (p < 0.05) differences between decoders.

Three variants of the general mirror decoder were built (*Active – Active*; *Active – Compliant; Compliant – Compliant*), which differed in the active or compliant condition of the calibration and testing datasets (see Fig. 3c). No significant differences between the CC values of the three decoders were found (p = 0.089). However, the *Active-Active* decoder outperformed the other two cases in terms of NRMSE (*Active-Compliant*: p = 0.002; *Compliant-Compliant*: p = 0.005). The error of the *Compliant-Compliant* case was also significantly (p = 0.049) lower than that of the *Active-Compliant* decoder (See Fig. 6).

### Proof of concept with chronic stroke patients

**Figure 7:**
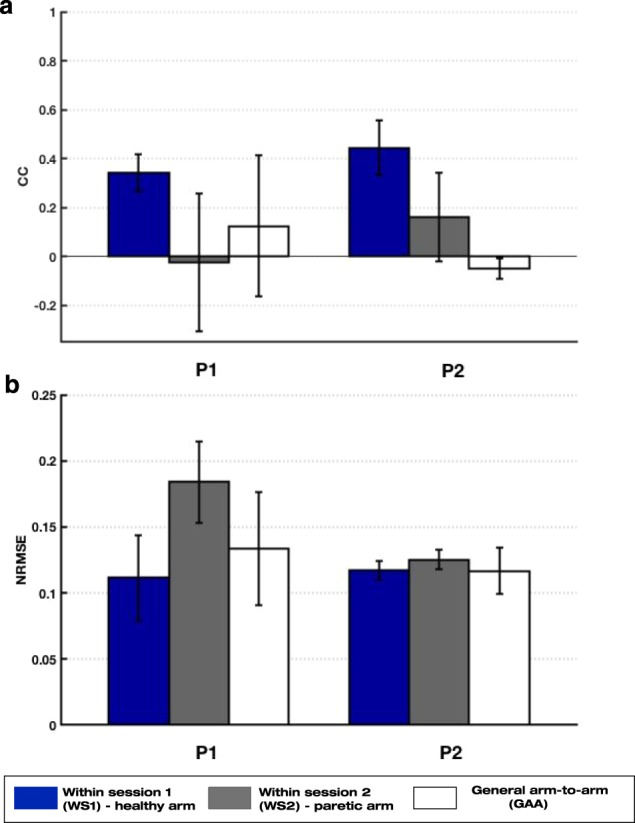
Performance values of the ipsilateral and mirror decoder from the experiment with chronic patients: (**a**) Correlation coefficient (CC) and **(b)** normalized root-mean-square error (NRMSE) mean and standard deviation values of the within-session decoder WS1 of the healthy arm (blue), the within-session decoder WS2 of the paretic arm (grey) and the general arm-to-arm GAA decoder (white) for P1 (left) and P2 (right) patients.

The effectiveness of the mirror decoder was evaluated in two chronic patients with moderate and severe paralysis. Both patients reported a good acceptance of the system, the exoskeleton ergonomics and mobility as well as the speed and complexity of the movements. Ipsilateral within-session decoders were also evaluated both for the healthy and paretic arms of each patient. Figure 7 illustrates the performance of the ipsilateral and mirror decoders for patients P1 (left; moderate impairment) and P2 (right; severe impairment). As expected, the within-session decoder WS1 of the healthy arm shows the highest performance values for both patients. We found variable and poor decoding performance for the within-session decoders WS2 of the paretic arm, as paretic EMG patterns are highly variable and abnormal. Moreover, it should be noticed that for P1 patient only 1 block of data of tasks T1 and T2 was recorded and thus, the 5-fold cross validation of WS2 was computed with little data. The values of the general arm-to-arm decoder (GAA) reflect a performance drop compared to WS1, due to the transfer across arms and the pathological EMG activity of stroke patients. Since the performance metrics show how similar the kinematics decoded from the paretic EMG are to the real kinematics determined by the assister, pathological EMG activity produces kinematics that deviate from the ideal trajectory and thus, lead to poor performance values. There is also a rather variable performance across DoFs for P1 compared to P2, as reflected in the larger standard deviation values. The correlation coefficient of patient P2 reached negative values due to the severe impairment and the existence of pathological muscle activations. However, these values are expected to raise as the patient learns the mirror mapping and the impairment level is reduced, as inferred from the higher CC values of patient P1 with moderate impairment. The mean error plot shows highest error values for the WS2 of the paretic arm and comparable values for the WS1 and GAA decoders for both patients.

## Discussion

In this study, we presented a novel rehabilitative concept that turns the focus of the existing myoelectric interfaces by enhancing the recovery of healthy muscle activation patterns. Furthermore, we tested and validated it on 8 healthy participants and 2 chronic stroke patients.

The significant performance difference between the within-session and the session-to-session decoders confirms that variables such as electrode position shift and impedance changes can affect the decoding accuracy. On the other hand, the difference between the session-to-session (SS) and the mirror decoders (TSAA and GAA) was not significant. This implies that the inter-limb variability of the EMG patterns is not big enough to produce a significant drop in the decoding performance, as suggested by previous studies^8,9^. This supports the use of the mirror decoder as a reference model for the paretic limb in the rehabilitation of stroke hemiplegic patients. Although a recalibrated decoder with data from the paretic arm could raise the decoding accuracy, this option was not considered since the aim of the mirror decoding paradigm is to impose the model of healthy activity on the paretic arm so that they can correct their pathological activity, instead of using it to decode their motion intention as accurately as possible.

The generalization analysis shows that the general decoder outperforms the task-specific one, especially when decoding EMG data from untrained tasks (TSAA-FM vs. GAA-FM). Utilizing a general decoder would avoid the need of switching between decoders depending on the task being executed at that moment. In addition, the general decoder would be advantageous to decode new tasks included in the course of the intervention or movements with a bigger range of motion, as the patient recovered certain motor function. On top of that, less data would be needed to calibrate a single general decoder than several task-specific decoders, as demonstrated by the analysis with equally balanced calibration datasets (TSAA-FM vs. GAA-FM). Therefore, it would be more practical and accurate to build a general decoder than various task-specific decoders. This knowledge was applied to further develop, optimize and test our platform in the experiment with stroke patients.

The decoder calibrated and tested under Active conditions (i.e. *Active –Active*) performed better than the other two cases. However, it should be noticed that the *Active-Active* decoder was trained with data from the fourth session of training with the dominant arm (S4) while the other decoders were trained with the first (S5) or second session (S6) of the non-dominant arm, which might have biased the results. Despite the higher performance of the *Active-Active decoder*, the possibility of employing such methodology with severely paralyzed patients is doubtful, as most of them would not be able to move the exoskeleton by themselves. Hence, considering patients’ impairment with a weak or atrophied musculature, the operation of the myoelectric interface with the paretic limb would have to be done under compliant conditions. Moreover, the movement of the exoskeleton is intended to be used as feedback for the patients to correct their paretic activity patterns. Therefore, the question is whether to calibrate the system with data from active (*Active - Compliant* case) or compliant movements (*Compliant - Compliant* case) with the healthy upper limb. On one hand, following and adapting to the pace and trajectory of the exoskeleton during a compliant movement may be challenging and the risk that the patients remain passive exists. That is why EMG should be continuously tracked and participants should be repetitively reminded that the movement had to be followed actively and as naturally as possible. The results show a lower error achieved by the *Compliant-Compliant* case over the *Active-Compliant* approach, indicating that if the conditions of the calibration and testing sessions are the same (*Compliant-Compliant*) the activation patterns might be more similar to each other. Moreover, performing the tasks with the healthy arm under compliant conditions may help the patient to get used to the pace and velocity profiles of the exoskeleton movements before the operation phase starts. Therefore, we propose using *Compliant* conditions during the calibration phase as a novel and optimal method to collect data to train a myoelectric decoder for rehabilitation therapies with stroke patients.

The presence of pathological muscle activity in chronic stroke patients is reflected in the poor correlation and large error values between the real recorded kinematics and the ones predicted from the EMG activity (Fig. 7, GAA decoder). As expected, the within-session decoder WS1 of the healthy arm of patients was the most accurate one, as this represents how good the decoder could estimate the kinematics without arm-to-arm, session-to-session or task-to-task variability. The transfer across arms, sessions and tasks, and the existence of pathological EMG activity resulted in a performance drop for the mirror decoder GAA. Although the number of channels for patient P1 was notably larger than for P2, the decoding performance of WS1 was lower for P1 than for P2, implying that the lower performance of the mirror decoder GAA for P2 compared to P1 might be mainly influenced by the severity of the impairment, measured by the clinical scales. Therefore, the higher the paresis and the spasticity level, the lower the EMG decoding performance values and the poorer the control. The lower performance achieved by the patients compared to the healthy participants supports this conclusion too (see Figs 4 and 7). From these results, one could infer that the modular organization of the muscle activity of the patient with moderate paralysis (P1) may resemble more that of a healthy individual, whereas rather pathological patterns could probably be found when looking at the EMG activity of patient P2, with severe paralysis. We anticipate that in a longitudinal study with severe patients, the initial performance will be poor and the real-time control unskilled. Alternatively, those patients with poor or no decodable muscle activity could initially train with an EEG-brain-machine-interface^48–52^ or a hybrid^47,53,54^ until they recovered sufficient EMG activity to benefit from a myoelectric therapy. We foresee that as patients train with this mirror myoelectric interface, the modular organization of the EMG activity will resemble more that of their healthy upper limb. Thus, the decoding performance and control of the exoskeleton would become more skillful and accurate, eventually leading to the recovery of certain degree of motor function. Nonetheless, in order to demonstrate such hypothesis, a longitudinal study including the assessment of the muscle synergy structure evolution and the functional impairment level along the intervention would be needed.

Overall, the proposed rehabilitation paradigm brings in several assets. First of all, it offers a well-founded^8,9^ method to promote the reintegration of healthy muscle activation patterns on the paretic limb of stroke patients, by utilizing the synergy structure of their intact upper limb as reference. Furthermore, this system is the first one that allows the simultaneous and continuous (direction and speed) myoelectric control of 7 DoFs of the upper limb, involving proximal and distal joints. This enables the training of functional multi-DoF movements of the upper limb in a synergistic fashion, which facilitates the translation of the re-learned motor skills to activities of daily living^5,55^. Additionally, it includes several features that are of paramount importance for the activation of neuroplastic mechanisms such as, closed-loop control with online contingent visual and proprioceptive feedback^48,49,51^, improved perception and constant active participation and engagement of the patient in the task^56,57^. Lastly, the majority of the stroke population could benefit from this type of therapy, since the only requirement is the presence of decodable EMG activity even in the complete absence of movement of the paretic limb, which has been found even in severely impaired patients^26^. The results presented here aided in the definition of certain aspects such as the calibration data conditions, and validated the effectiveness of the system in chronic stroke patients. Therefore, we envisage this approach to be a potential rehabilitation method to elicit the recovery of healthy muscle recruitment patterns in stroke patients of a wide range of impairment levels. Nonetheless, further developments such as the implementation of synergy-based algorithms that have been reported reliable and robust^58,59^ could be implemented to boost the decoding performance and to ensure that different muscle activation patterns do not lead to the same kinematics. In the future, a longitudinal study that includes the real-time operation of the interface by stroke patients should be carried out in order to assess the rehabilitation effects of the proposed method.

## Data Availability

All the materials, data, code, and associated protocols are available to the readers.
